# A perfect storm: examining the synergistic effects of negative and positive emotional instability on promoting weight loss activities in anorexia nervosa

**DOI:** 10.3389/fpsyg.2015.01260

**Published:** 2015-08-31

**Authors:** Edward A. Selby, Talea Cornelius, Kara B. Fehling, Amy Kranzler, Emily A. Panza, Jason M. Lavender, Stephen A. Wonderlich, Ross D. Crosby, Scott G. Engel, James E. Mitchell, Scott J. Crow, Carol B. Peterson, Daniel Le Grange

**Affiliations:** ^1^Department of Psychology, Rutgers University, Piscataway, NJ, USA; ^2^Department of Psychology, University of Connecticut, Storrs, CT, USA; ^3^Neuropsychiatric Research Institute, Fargo, ND, USA; ^4^School of Medicine, University of North Dakota, Bismarck, ND, USA; ^5^Department of Psychiatry, University of Minnesota, Minneapolis, MN, USA; ^6^Department of Psychiatry and Behavioral Neuroscience, University of Chicago, Chicago, IL, USA

**Keywords:** anorexia nervosa, positive emotion, emotional instability, purging, weighing

## Abstract

Growing evidence indicates that both positive and negative emotion potentially influence the development and maintenance of anorexia nervosa, through both positive and negative reinforcement of weight loss activities. Such reactive emotional experience may be characterized by frequent and intense fluctuations in emotion, a construct known as “emotional instability.” The purpose of this study was to investigate the association between positive emotional instability and weight loss activities in anorexia nervosa, and to investigate the synergistic effects of positive and negative emotional instability on promoting weight loss activities. Using ecological momentary assessment methods, 118 participants with anorexia nervosa reported their emotional experiences and behaviors at least six times daily over 2 weeks using a portable digital device. Using generalized linear modeling, results indicated that high levels of both positive and negative emotional instability, and the interaction between the two, were associated with more frequent weight-loss activities, beyond anorexia subtype and mean levels of emotional intensity. These findings indicate that when women with anorexia exhibit both high levels of both positive and negative emotional instability they are more prone to a variety of weight loss activities. The importance of addressing the role of both positive and negative emotion in anorexia treatment is discussed.

## Introduction

Anorexia nervosa is a disorder characterized by refusal to maintain normal weight, deliberate caloric restriction, and persistent behaviors that interfere with weight gain (American Psychiatric Association (APA), 2013). Anorexia has a high mortality index (Papadopoulos et al., 2009) and suicide rate (Selby et al., 2010), and is difficult to treat (Vandereycken, 2006). Pervasive negative emotion typically characterizes the anorexic experience (Engel et al., 2013), likely contributing to the treatment resistance of anorexia. Thus, weight-loss behaviors may be driven by these negative emotions, as these behaviors can function to reduce negative emotion arising from body and weight/shape concerns (Smyth et al., 2007), creating a cycle of negative reinforcement. Although the role of dysregulated negative emotion in the genesis and maintenance of weight-loss behaviors in anorexia is well established, the potential role of dysregulated positive emotion is not. Expanding upon growing evidence that positive emotion is dysregulated in anorexia, the current study examines the potential synergistic effects of dysregulated negative and positive emotion in maintaining weight-loss behaviors in anorexia.

### Dysregulated Positive Emotion in Anorexia Nervosa

While most theoretical approaches to anorexia have highlighted and described the role of negative emotion in detail (Slade, 1982; Fairburn et al., 2003), these models have traditionally neglected the role of positive emotion. Burgeoning research, however, suggests that dysregulated positive emotion can be maladaptive (Gruber et al., 2011). Only recently has more emphasis been placed on the potential role of positive emotion in anorexia, with increasing focus on interpersonal and intrapersonal reinforcements experienced in the early stages of the disorder (Walsh, 2013). Qualitative studies provide further information about the potential role of positive emotions in anorexia, particularly investigation of the “Pro-Anorexia” Internet phenomenon, which features images of emaciated women, inspirational weight loss quotes, and promotion of anorexic behaviors (Juarascio et al., 2010). Quotes from case studies on anorexia also provide evidence of maladaptive positive emotions and cognitions regarding weight loss behavior, with one patient noting that to be thin resulted in a “sense of pride, power, and accomplishment” (Bruch, 1978), and another stating, “While I was anorexic, I had always considered myself to be extraordinary rather than abnormal” (MacLeod, 1982). Furthermore, for many people with anorexia, losing weight makes them feel more attractive, builds self-control, helps them feel physically fit, provides feelings of confidence, makes them feel able to do at least one thing better than other people, enhances feelings of expertise, and improves their ability to push their body further (Serpell et al., 1999, 2004).

Recent empirical studies have more thoroughly explored the role of maladaptive positive emotion in anorexia. In a recent study, those with anorexia nervosa who had difficulty distinguishing between discrete positive emotions also tended to report increased positive emotion in response to, and as a result of, more weight loss activities such as: restricting, self-induced vomiting, laxative misuse, exercise, and self-evaluation activities, such as weighing and checking body fat (Selby et al., 2014). Women with anorexia have also been found to have stronger implicit associations between images of emaciated women and words like “beauty” than control participants (Smith et al., 2014). Biological studies have also uncovered evidence of maladaptive responses to reward in anorexia, a biological index of positive emotion (Park et al., 2014). For example, neuroimaging studies have demonstrated that women with anorexia may be more neurologically responsive to reward anticipation and reception than normal weight or overweight controls, as indicated by increased activation of brain dopamine reward circuits in response to food stimuli (Frank et al., 2012). Similarly, women with anorexia also demonstrate increased ventral striatal reward responsiveness, the same region of the brain that has been found to activate in response to consumption of addictive substances, to images of underweight women (Fladung et al., 2010). Thus, increasing evidence that dysregulated positive emotion is involved in anorexia only emphasizes the need for research to further clarify this association.

### Emotional Instability and Anorexia

One potential reason that dysregulated positive emotion in anorexia nervosa has been understudied may involve the way positive and negative emotion have historically been conceptualized. Negative emotion, for example, has often has been conceptualized as a “trait” characteristic, with consistent and chronic elevations considered problematic, as in the case of depression (Watson et al., 1988a). Similarly, positive emotion has also been predominantly conceptualized as a trait (Watson et al., 1988a), considered maladaptive primarily when it is consistently elevated to an excessive extent, as in the case of full-blown manic episodes, or excessively and chronically low, as in the case of depression (e.g., anhedonia). Along these lines, anorexia involves pervasive and chronic negative emotion (Engel et al., 2013), and has been associated with *low* mean levels of positive emotion intensity (Pryor and Wiederman, 1996; Selby et al., 2014). Yet, the conceptualization of positive emotion being primarily absent in anorexia is problematic, as women with anorexia do not necessarily appear anhedonic and, in contrast, are often working quite hard at weight loss activities and frequently report being inspired by comments from others about improvements in physical appearance after initial weight loss in the early stages of the disorder (Walsh, 2013).

Importantly, conceptualizations of dysregulated emotion have broadened over recent years to include not only cases of chronic elevations in or a dearth of emotion (both positive and negative), but now can also be represented by frequent fluctuations in emotion intensity—a construct known as “emotional instability” (Trull et al., 2008). Emotional instability, in contrast to a consistent or stable emotional state/mood, is defined as frequent and intense fluctuations in emotion over the course of a short period of time (Jahng et al., 2008), where an individual with unstable emotions may report multiple elevations and decreases in emotions within the course of even 1 day. Emotional instability represents a distinct problem in the experience of emotion that extends beyond the traditional focus on chronic and stable emotional extremes, and instead represents more of an emotional “reactivity” to one’s environment, thoughts, perceptions, and behavior. Although emotional instability can also involve extreme elevations in emotion, these elevations are transient and frequently fluctuating from baseline to extreme elevations multiple times throughout the day (Trull et al., 2008).

Negative emotional instability has been linked to multiple psychopathologies, including bulimia nervosa (Selby et al., 2012) and borderline personality disorder (Selby and Joiner, 2009). Similarly, negative emotional instability has also been indicated as a problem in anorexia and is associated with baseline eating disorder symptoms and dietary restriction (Lavender et al., 2013). Only recently, however, has positive emotional instability been studied empirically, and it has not yet been investigated in samples of individuals with eating disorders. For example, in one undergraduate sample, Hill and Updegraff (2012) found that variability in positive emotion was associated with elevated positive emotional reactivity. Growing evidence indicates that positive emotional instability, despite being associated with increased positive emotion, is not always a good thing. In another non-clinical sample, Gruber et al. (2013) also reported that extreme variability of positive emotion was associated with worse psychological health, lower life satisfaction, and decreased happiness. This line of research is consistent with findings that heart rate variability, a parasympathetic index of positive emotion, may have a curvilinear relationship with well-being, indicating that higher levels of heart rate variability (and therefore positive emotion) may be associated with worse mental health (Kogan et al., 2013).

In the case of anorexia, positive emotional instability may be more problematic than chronic low mean-level positive emotional intensity. Mean-level positive emotion intensity actually tends to be negatively associated with most weight-loss behaviors (Selby et al., 2014), whereas positive emotional instability may be positively associated with weight-loss behaviors and social positive reinforcement (Walsh, 2013; Selby et al., 2014). Accordingly, high instability of positive emotion in anorexia may be due to the interplay between positive emotion and weight loss behaviors, wherein positive emotion serves to reinforce successful weight loss behaviors and success with weight loss serves to motivation further weight loss behaviors (Selby et al., 2014). As a result, positive emotion may fluctuate drastically in those with anorexia nervosa, either as a result of weight loss efforts, in motivation for further weight loss efforts, or in response to environmental reinforcement of weight loss activities (Walsh, 2013). Thus, positive emotional instability may have a positive association with frequency of weight loss behaviors, and these issues highlight the necessary distinction between mean-level positive emotional intensity and the frequent and intense fluctuations of positive emotional experience characterized by emotional instability.

### Synergistic Effects of Positive and Negative Emotional Instability

Increasing evidence suggests that the emotional experiences of those with anorexia may be characterized both by pervasively *dysregulated* negative *and* positive emotion (Lavender et al., 2013; Selby et al., 2014). Weight-loss behaviors may be both positively reinforced (through positive emotion arising from successful weight loss or self-control) and negatively reinforced (through alleviation of negative emotions arising from body image and weight-gain concerns). While these findings may seem contradictory, empirical research indicates that positive and negative emotion may be more accurately described as bivariate rather than existing on a unipolar scale (Larsen et al., 2001)—that is, they are not mutually exclusive, and can be experienced simultaneously. In anorexia, the relationship between emotions and eating disorder symptoms may be best represented as an interaction between negative and positive emotional reinforcement, wherein weight loss behaviors are positively reinforced with the progression of weight loss, and those same behaviors are negatively reinforcing when weight loss plateau or weight is gained, or concerns with body image or shape arise. Accordingly, both positive and negative emotional responses are likely to fluctuate frequently as a function of success or difficulty with weight loss goals and perceptions, and outcome that may be measured through the assessment of negative and positive emotional instability. Thus, the synergistic effects of both positive and negative forces may result in a “perfect storm” of motivation for weight-loss activity, and may, in conjunction with biological and other risk factors, lead to the extreme weight loss that characterizes anorexia nervosa.

### Ecological Momentary Assessment

One way to expand our understanding of the association between negative and positive emotional experiences and weight loss behaviors in anorexia nervosa is through the use of ecological momentary assessment (EMA) methods. EMA methods are a set of relatively new research techniques that have emerged over the last two decades, in which participants record emotional and behavioral experiences multiple times daily over a period of time, frequently using digital devices such as palm pilots or smartphones. EMA provides a number of advantages to traditional research methods, which often rely on single point assessments that utilize retrospective recall of emotional experience and behavior. While there are many merits to retrospective assessments, EMA reduces retrospective recall by asking participants to rate various experiences multiple times each day, allowing for more accurate and precise reporting, particularly in the case of eating disordered behaviors (Smyth et al., 2001). EMA allows for more precise measurement of variables that are known to fluctuate over the course of a day or multiple days, such as emotional experience (Trull et al., 2008). For example, using EMA methods Engel et al. (2013) found that increased negative emotion was predictive of dietary restriction in a sample of women with anorexia, and negative emotion was found to increase prior to and substantially decrease after eating disorders behaviors occurred.

Ecological momentary assessment methods are also provide an improved method for measuring emotional instability, as opposed to retrospective self-report measures that ask participants to estimate how reactive their emotions are (Anestis et al., 2010; Lavender et al., 2013). Indeed, with EMA methods research can derive emotional instability using formulas [such as the mean squared successive deviation (MSSD) equation] that aggregate multiple participant assessments of various emotions reported multiple times each day by each participant, creating one index that provides an empirical estimate of emotional instability. Instability indices examine temporally chained observations and take into account shifts in the magnitude of emotion from one time point to a subsequent time point (Ebner-Priemer et al., 2007). Thus, someone with an elevated instability index experiences emotions that rise and fall frequently. In contrast, an individual who consistently reports extremely high levels of negative emotion would be considered to have low emotional instability, as they experience little variability in the magnitude of their emotions. Using such methods, previous EMA derived instability indices of negative emotion have been linked to weight loss behaviors in a sample of women with anorexia nervosa (Lavender et al., 2013). However, no studies have investigated the role of EMA derived positive emotion instability indices in the weight loss behaviors of women with anorexia nervosa, nor have they examined a potential interaction between negative and positive emotional instability in predicting even more weight loss behaviors than either instability index alone.

### Current Study

The purpose of the current study was to further investigate emotional instability and its association with weight loss behaviors in anorexia nervosa using data from one of the largest EMA studies on anorexia nervosa completed to date (Engel et al., 2013; Selby et al., 2014). Using EMA methods, the current study builds on the previous findings of Lavender et al. (2013), who demonstrated negative emotional instability was associated with more frequent weight loss activities, and the recent findings by Selby et al. (2014), who found that poor understanding of positive emotions predicted increased weight loss activities, by examining the association between positive emotional instability and weight loss activities in a sample of women diagnosed with anorexia nervosa. Using an EMA derived measure of positive emotional instability, we examined the association between positive emotional instability and self-induced vomiting, laxative misuse, exercise, weighing, checking for body fat, and calorie restriction, over and above any mean level effects of negative and positive emotional intensity. Furthermore, because no studies to our knowledge have examined the synergistic effects of positive and negative emotion on weight loss behaviors in anorexia, we also examine the potential for an interaction effect between negative and positive emotional instability in predicting reports of more frequent weight loss behaviors.

We hypothesized that increased positive emotional instability would be associated with more frequent reports of all weight loss activities recorded during the 2-week EMA protocol. Furthermore, because negative emotional instability has been also been linked to weight loss activities in anorexia nervosa (Engel et al., 2013; Lavender et al., 2013), we hypothesized that there would be an interaction between EMA derived indices for negative emotional instability and positive emotional instability, such that those with elevated instability indices for both positive and negative emotion would also exhibit the highest reports of weight loss activities.

## Materials and Methods

### Participants

Between 2007 and 2010, 118 women who met current diagnostic or subclinical criteria for anorexia (as defined below), with designation as either restricting [*N* = 73] or binge-purge subtype [*N* = 45] participated in this study. All participants completed an EMA protocol. 601 potential participants were originally screened for eligibility via phone. Eligibility criteria for further participation in the study included: (1) female sex, (2) at least 18 years old, and (3) at least subthreshold criteria for anorexia. Diagnoses for anorexia were modified to include the following subthreshold exceptions: (1) body mass index between 17.5 and 18.5, or (2) absence of amenorrhea or an absence of the cognitive features of anorexia (Engel et al., 2013). These modifications are consistent with changes to DSM-5 criteria for anorexia (Mitchell et al., 2005; American Psychiatric Association (APA), 2013), which has removed the amenorrhea criterion. More details on participant recruitment can be found in Engel et al. (2013). Participants’ ages ranged from 18 to 58 years (*M* = 25.3, SD = 8.4 years) and had an average body mass index of 17.2 kg/m^2^ (SD = 1.0; range = 13.4–18.5 kg/m^2^). The sample was almost entirely non-Hispanic Caucasian (96.6%), with some African American (2.3%) and Asian American (1.1%) participants.

### Procedures

Participant recruitment transpired at three coordinated sites (Fargo, ND, USA; Minneapolis, MN, USA; and Chicago, IL, USA) through mailings to treatment professionals, on-line postings, advertisements in community and campus newspapers, and flyers posted in clinical, community and campus settings. Following the phone screening, participants were scheduled for two in-person clinical assessment visits. During these visits, the research staff performed physical screening and laboratory tests were to ensure medical stability and conducted structured clinical interviews to confirm diagnosis. Research staff then trained participants on the use of a digital recording device (Handspring Visor)^1^ used for EMA. In order to capture a variety of mood ratings in close temporal proximity to the behaviors, signal-contingent, event-contingent, and interval-contingent recordings were employed (Wheeler and Reis, 1991). For each type of recording, participants were asked to complete ratings of their mood and behaviors. Signal-contingent recordings prompted participants to complete mood ratings six times throughout the day when the digital recording device signaled them. The device signaled participants at semi-random times within 30–45 min of the anchor times: 8:30 am, 11:10 am, 1:50 pm, 4:30 pm, 7:10 pm, and 9:50 pm. Event-contingent recording was employed as participants were asked to complete ratings whenever they engaged in certain eating disordered behaviors. Participants also completed interval-contingent reports at the end of the day before going to sleep.

Participants carried the EMA device for two practice days, then returned and provided the data recorded during their practice period (practice data were not used in analyses.) This practice period was used both to ensure participants were familiar and comfortable with the EMA assessments and to minimize reactivity to the recording procedures [although there is little evidence of reactivity in EMA (Stein and Corte, 2003)]. The data from the practice days were reviewed, and participants were given feedback regarding their compliance rates to random device notifications. Participants were then given the digital recording device to complete EMA recordings over the next 2 weeks. Attempts were made to schedule 2–3 visits for each participant during this 2-week interval to obtain recorded data and to minimize the amount of data lost in the event of technical problems. At each visit participants were given feedback on their compliance rates. The end of the 2-week monitoring period concluded the participants’ study participation. Participants were compensated $100 per week for their participation and were given a $50 bonus for a compliance rate of at least 80% to random signals.

The independent ethics review committees of each site approved this study. All participants were fully informed about the study and the study procedures by research staff at each site. All participants provided written informed consent.

### Clinical Assessment

The *Structured Clinical Interview for DSM-IV Axis I Disorders* (SCID-I/P; First et al., 1995) was used for determining anorexia nervosa diagnoses at the full and subthreshold level. A doctoral-level clinical psychologist completed the clinical interviews. All interviews were recorded, and a second independent assessor, blinded to the original diagnosis, rated current eating disorder diagnoses in a random sample of 25% of the total sample (*n* = 30). Interrater reliability for current anorexia diagnosis was found to be very good (Kappa = 0.93).

### Ecological Momentary Assessment

For all random and self-prompted assessments, participants recorded their current emotions and weight-loss activities or caloric restriction since the previous recording.

#### Positive and Negative Emotion Measures

These items were used to generate indices of average positive and negative emotion, as well as instability indices for these emotional valences. At each of the momentary assessments participants answered questions about specific, current emotions on a scale ranging from (1) *not at all* to (5) *extremely*. Mood items were generated from the *Positive and Negative Affect Scale* (PANAS; Watson et al., 1988b) and the *Profile of Mood States* (POMS; Lorr and McNair, 1971). Items from the PANAS included eight negative emotions (NEs; nervous, angry at self, afraid, sad, disgusted, distressed, ashamed, and dissatisfied with self) and eight positive emotions (PEs; strong, enthusiastic, happy, energetic, proud, attentive, confident, and cheerful). Seven items from the tension/anxiety scale of the POMS (on edge, restless, tense, anxious, uneasy, shaky, and panicky) were also used for NE, and one positive item from the POMS (relaxed) was included as a PE item. In the current study, alpha coefficients for NE and PE were 0.94 and 0.92 respectively.

#### Weight-Loss and Evaluation Behaviors

Participants completed a checklist of common weight-loss activities at each momentary assessment. This behavioral checklist has been used extensively in previous EMA research on eating disordered and weight loss behaviors (Smyth et al., 2007; Engel et al., 2013; Selby et al., 2014). These activities included self-induced vomiting or laxative misuse for weight control, weighing-in on a scale, exercising, and checking joints and bones for fat. All participants were given clarification regarding the definitions of each activity during EMA training.

#### Calorie Restriction

Participants were asked to report the occurrence of specific eating-related behaviors and rituals using questions drawn from the *Yale-Brown-Cornell Eating Disorder Scale* (YBC-EDS; Sunday et al., 1995). At the end-of-day assessment participants were asked if they had limited their food intake to less than 1200 Kcal during that day. During training, participants were given examples of objective amounts of food consumed to help them improve accuracy of calorie intake estimation. This dichotomous variable was later summed across all daily observations for the study to generate a count variable for number of days out of 14 with calorie restriction.

### Data Analytic Strategy

In order to examine the effects of emotional instability on weight-loss activities, we created instability indices for both positive and negative emotion. To calculate the instability indices we used the adjusted successive difference (ASD; Jahng et al., 2008) correction to the MSSD equation (von Neumann et al., 1941), which is displayed below:

(1) $$MSSD\left(X\right)=\frac{\sum_{i=2}^{n}{\left(x_{i}-x_{i-1}\right)}^{2}}{n-1}$$

The MSSD equation takes into account both the amplitude and frequency of the variable (*x*), where *x* in this equation pertained to momentary ratings of either positive or negative emotion, *i* pertained to each individual recording emotions, and *n* pertained to the number of observations for each individual. The ASD improves upon the MSSD instability index by accounting for the frequency and intensity of individual changes in the variable from one recording to the next as well as the time interval difference between random signals (Jahng et al., 2008). The ASD index is particularly useful for measuring short-term, successive change within a single day. The ASD equation is displayed below:

(2) $${\text{ASD}}_{i+1}=\frac{x_{i+1}-x_{i}}{{\left[\left(t_{i+1}-t_{i}\right)/mdn\left(t_{i+1}-t_{i}\right)\right]}^{\lambda }}$$

In the ASD equation, momentary changes in the variable (*x*) are a function of the difference in *x* from one occasion (*i*) to the next, divided by the quotient of the difference in time interval (*t*) from one signal to the next, divided by the median (*Mdn*) of *t* for all observations. Dividing the time interval by its median results in a rescaled median of 1. The median rescaling of time is then raised to the λ power, where λ refers to the serial autocorrelation between changes in *x* and *t*. Possible λ values range from 1 for high serial correlations, to 0 indicating no serial correlation, to –1 for an inverse serial correlation. The outcome of the ASD index is then used to adjust the MSSD by replacing ($x_{i}-\bar{x}$) with ASD_*i* + 1._ In the current sample, the autocorrelation index (and thus λ) for momentary positive emotion was –0.064 (*p* < 0.05), and for negative emotion it was 0.024 (*p* < 0.05), indicating minor adjustments were needed for both variables.

In addition to emotional instability indices, we created variables for mean levels of positive and negative emotional intensity over the duration of the study for each participant by averaging all respective recordings for that participant. Weight-loss behaviors were aggregated across the monitoring period for each participant (e.g., total number of exercise sessions, laxative misuses, etc.). We also created a variable representing the total number of all weight-loss activities reported by each individual over the course of the study; all weight-loss activities were *z*-scored to ensure that all contributed equally to the total variable.

In order to examine the interaction between positive and negative emotional instability on weight-loss activities, we used generalized linear modeling to account for the non-normal distribution of the activity recordings, which were count variables. A log link function with a Poisson distribution was used accordingly, to address the non-normality of data. All Poisson analyses report the relative risk ratio (RR) effect size statistic, which can be interpreted as the amount of increase in the outcome variable for every one-unit increase in the independent variable. First, we examined the main effects of mean negative and positive emotional intensity and negative and positive emotional instability. Next, the interaction between positive and negative emotional instability was entered into the regression equation to test significance beyond any main effects. The analysis examining the total *z*-scored behaviors variable used linear regression, as the *z*-score transformations were not integers, which are required for Poisson analysis. Finally, we re-examined models with anorexia subtype as a covariate, as anorexia binge-purge subtype tends to be associated with higher rates of some weight-loss and evaluation behaviors such as vomiting and laxative misuse.

## Results

### Preliminary Analyses

The 118 participants in this study provided a total of 15,017 momentary recordings during the monitoring period, representing 1,767 separate participant days. Recordings included 3,445 reports of eating-relevant events, with Table 1 displaying an average of 4.53 vomits (SD = 9.39), 0.62 misuses (SD = 1.93), 6.59 exercise sessions (SD = 8.59)^2^, 5.33 weigh-ins (SD = 7.62), 20.61 checks for body fat (SD = 26.62), and 4.03 days with under 1200 calories consumed (SD = 5.22). Compliance rates to signals averaged 87% (range = 58–100%); 77% of all signals were responded to within 45 min. Compliance with end-of-day ratings averaged 89%. Positive emotion scores were higher in those with anorexia restricting subtype (*M* = 18.79, SD = 6.77) than those with binge-purge subtype [*M* = 17.56, SD = 6.98; *t*(117) = 10.46, *p* < 0.001, *d* = 0.17]. Conversely, those with binge-purge subtype had significantly higher levels of positive emotional instability (*M* = 45.65, SD = 28.56) than those with restricting subtype [*M* = 42.33, SD = 30.58; *t*(117) = 6.61, *p* < 0.001; *d* = 0.31]. Mean negative emotion scores were higher in those with binge-purge subtype (*M* = 20.93, SD = 8.00) than restricting subtype [*M* = 16.83, SD = 6.65; *t*(117) = 3.00, *p* < 0.05; *d* = 0.56]. Similarly, those with binge-purge subtype demonstrated higher negative emotional instability (*M* = 384.96, SD = 242.25) than those with restricting subtype [*M* = 252.63, SD = 180.10; *t*(117) = 3.39, *p* < 0.001; *d* = 0.62].

**TABLE 1:** **Means and standard deviations for key variables**.

	***M***	**SD**	**MIN**	**MAX**
1. Average EMA negative emotion level	34.56	13.24	16.00	73.00
2. EMA negative emotional instability	300.87	214.74	51.91	1023.21
3. Average EMA positive emotion level	20.79	5.71	9.00	39.00
4. EMA positive emotional instability	43.02	29.35	1.92	146.35
5. Vomiting episodes	4.53	9.39	0.00	43.00
6. Laxative misuses	0.62	9.93	0.00	13.00
7. Exercise sessions	6.59	8.59	0.00	47.00
8. Weigh-ins	5.33	7.62	0.00	52.00
9. Body fat checks	20.61	26.62	0.00	88.00
10. Days <1200 calories	4.03	5.21	0.00	14.00

N = 118. EMA, ecological momentary assessment derived variable; Days <1200 calories, days where caloric intake was restricted to be below 1200 calories.

### Main Effects

Regarding mean level emotion intensity (see Table 2), findings were consistent with past literature, with high levels of negative emotion and low levels of positive emotion being associated with more weight-loss activities. The only exception to this was exercising, which was associated with increased mean positive emotion and decreased mean negative emotion. Main effects for negative and positive emotion instability indicated that high levels of both were associated with more weight-loss activities, supporting our first hypothesis that elevations in positive emotional instability would be associated with more frequent weight loss activities.

**TABLE 2:** **Predicting overall frequency of weight loss and evaluation behaviors in anorexia**.

**Outcome**	**Predictor**	***B***	**SE**	***t*(113–114)**	***p***	**RR**
**Vomits**
	PE mean level	–0.454	0.025	–17.89	<0.001	0.635
	NE mean level	0.194	0.018	10.73	<0.001	1.214
	PE instability	0.081	0.004	17.78	<0.001	1.008
	NE instability	0.005	0.001	4.52	<0.001	1.005
	PE × NE instability	–0.0001	0.00001	–10.17	<0.001	0.999
**Laxative misuses**
	PE mean level	–0.037	0.011	–3.34	0.001	0.964
	NE mean level	0.086	0.009	10.04	<0.001	1.089
	PE instability	0.008	0.002	3.74	<0.001	1.008
	NE instability	0.002	0.001	3.42	0.001	1.002
	PE × NE instability	0.00008	0.000003	4.98	<0.001	1.0001
**Exercise sessions**
	PE mean level	0.060	0.023	2.46	0.014	1.062
	NE mean level	–0.083	0.016	–5.07	<0.001	0.920
	PE instability	0.071	0.004	17.31	<0.001	1.074
	NE instability	0.009	0.001	9.44	<0.001	1.009
	PE × NE instability	0.0001	0.00001	5.94	<0.001	1.0001
**Weigh-ins**
	PE mean level	–0.048	0.020	–2.41	0.016	0.610
	NE mean level	0.576	0.014	40.59	<0.001	1.78
	PE instability	0.002	0.003	0.51	0.611	1.002
	NE instability	0.001	0.0002	4.45	<0.001	1.001
	PE × NE instability	0.0001	0.00001	8.27	<0.001	1.0001
**Checking joints and bones for fat**
	PE mean level	–0.064	0.069	–0.92	0.359	0.938
	NE mean level	1.76	0.049	35.38	<0.001	5.812
	PE instability	0.048	0.012	3.87	<0.001	1.049
	NE instability	0.081	0.003	26.41	<0.001	1.084
	PE × NE instability	0.0002	0.00004	5.36	<0.001	1.0002
**<1200 Calories over 24 h**
	PE mean level	–0.17	0.040	–19.42	<0.001	0.843
	NE mean level	0.095	0.028	11.47	0.001	1.100
	PE instability	0.021	0.008	6.65	0.010	1.021
	NE instability	0.005	0.002	7.53	0.006	1.005
	PE × NE instability	0.00005	0.00002	5.87	0.015	1.0001
**Total weight loss activities (*z*-scored)**
	PE mean level	–0.12	0.007	–15.72	<0.001	.887
	NE mean level	0.21	0.005	38.27	<0.001	1.234
	PE instability	0.025	0.001	17.95	<0.001	1.025
	NE instability	0.009	0.0003	26.35	<0.001	1.009
	PE × NE instability	0.00004	0.000005	8.65	<0.001	1.0001

### Interaction Between Instability of Positive and Negative Emotion

Interaction analyses between the indices of instability of both negative and positive emotion were conducted to determine if increased weight-loss behaviors were better predicted by an interactive or an additive model, and to determine if this interaction was significant beyond main effects. The results of these analyses are detailed in Table 2.

**FIGURE 1:**
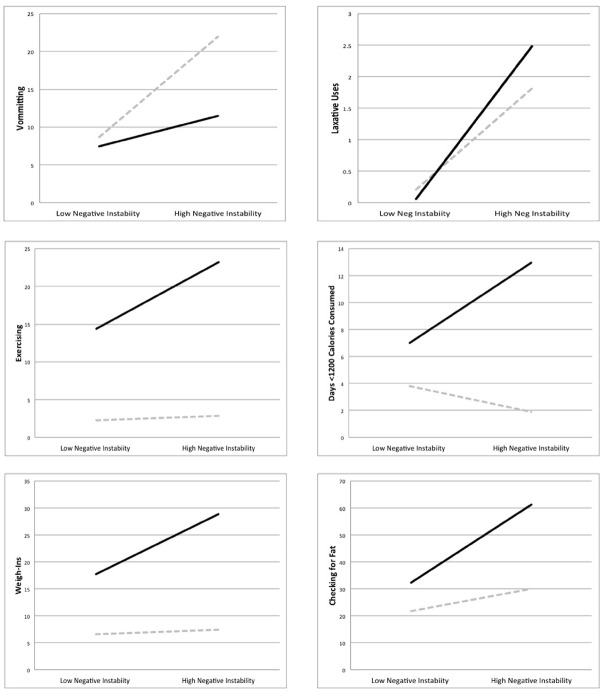
**Interaction between positive and negative emotional instability predicting increased weight loss activities in a sample of those with anorexia nervosa.** High and low values represent two standard deviations above and below the mean. Solid dark lines represent high positive emotional instability, while dashed gray lines indicate low positive emotional instability.

**FIGURE 2:**
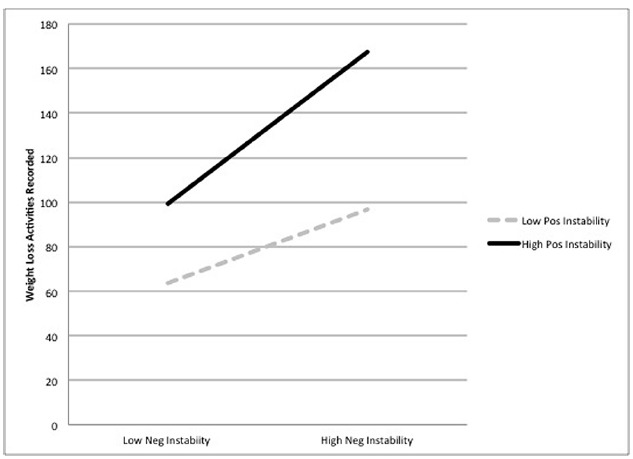
**Interaction between positive and negative emotional instability predicting total weight loss activities (***z***-scored).** High and low values represent two standard deviations above and below the mean. Solid dark lines represent high positive emotional instability, while dashed gray lines indicate low positive emotional instability.

The interaction between negative and positive emotional instability was significant for all weight-loss activities, and the graphs of these interactions are displayed in Figure 1. The combination of both high levels of negative and positive emotional instability predicted the highest levels of weight-loss behaviors, including laxative misuse [*B* = 0.00008, SE = 0.000003, *t*(114) = 4.98, *p* < 0.001, RR = 1.0001]^3^, exercising [*B* = 0.0001, SE = 0.00001, *t*(114) = 5.94, *p* < 0.001, RR = 1.0001], checking for fat [*B* = 0.0002, SE = 0.00004, *t*(114) = 5.36, *p* < 0.001, RR = 1.0002], weighing [*B* = 0.0001, SE = 0.00001, *t*(114) = 8.27, *p* < 0.001, RR = 1.0001], and restricting [*B* = 0.00005, SE = 0.00002, *t*(114) = 5.87, *p* = 0.015, RR = 1.0001]. Although the interaction for vomiting was significant [*B* = –0.0001, SE = 0.00001, *t*(114) = –10.17, *p* < 0.001, RR = 0.999], graphing out this interaction indicated that those with high negative emotional instability and low positive emotional instability reported the highest number of vomits. Finally, we examined a *z*-scored composite variable which took into account total frequency of all weight loss behaviors reported during the study with the interaction between positive and negative emotional instability. This interaction was significant [*B* = 0.00003, SE = 0.00001, *t*(114) = 5.22, *p* < 0.001, RR = 1.0001], and as indicated in Figure 2, participants reporting high levels of both positive and negative emotional instability also reported the most weight loss behaviors during the study. All interaction analyses were re-examined with anorexia subtype included as a covariate. Although anorexia binge-purge subtype was associated with elevated levels of some behaviors, all interaction terms remained significant.

**FIGURE 3:**
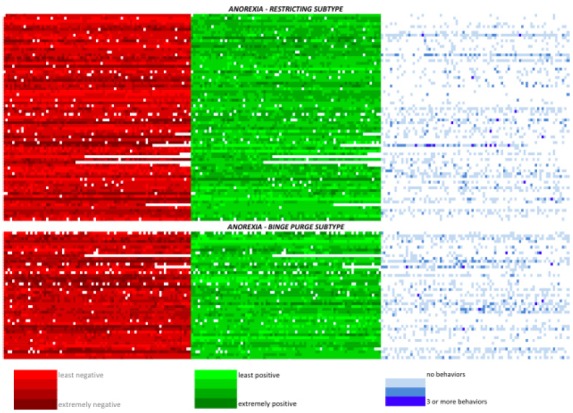
**Color-coded representation of participants’ emotional and behavioral experience at each signal recording during the monitoring period, group by anorexia subtype.** One horizontal line reflects one participant’s recordings. Dark red and green indicate elevations (two standard deviations) above the mean in negative and positive emotion, respectively. For the negative and positive emotion lines, white squares indicate non-completed assessments. Light blue squares indicate when a single weight loss activity was reported; dark blue squares represent three or more activities reported at that signal.

In order to obtain a qualitative representation of emotional fluctuations for participants across the duration of monitoring, we generated a color-coded figure where each signal was coded for each participant across the duration of monitoring (see Figure 3). Light red and green boxes indicate low levels (one standard deviation below average) of negative and positive emotion, respectively, and dark red and green boxes indicate elevated levels of positive and negative emotion (one standard deviation above average), respectively. This figure also contains blue squares, the darker of which indicate signals where one or more weight loss behaviors was engaged in. As can be seen in this figure, many participants demonstrated frequent and intense fluctuations in both negative and positive emotion, as well as variation in number of behaviors reported.

## Discussion

Although many studies have examined the role of negative emotion in facilitating weight-loss behaviors in anorexia, few studies have examined the potential role of positive emotion (Selby et al., 2014), and to our knowledge none have examined patterns of emotional instability in anorexia nervosa. In this study we examined the interaction between positive and negative emotional instability in terms of relationship to weight-loss behaviors in anorexia. EMA results indicated that anorexia patients who exhibited higher levels of positive emotional instability reported more frequent laxative misuse, exercise sessions, weigh-ins, checks for body fat, and restricting days. Furthermore, a significant interaction was supported such that those with anorexia who reported higher levels of both negative and positive emotional instability also reported the most frequent weight loss activities. These effects held even when controlling for mean levels of negative and positive emotional intensity and for anorexia subtype. However, only partial support was found for vomiting, although negative emotional instability had a stronger influence on this variable than positive emotional instability.

This study is the first to look at both positive emotional instability and the combined effects of positive and negative emotional instability on weight-loss behaviors in anorexia. Results suggest that both may play important roles in the development and maintenance of the disorder. For example, on occasions where a patient meets her weight-loss goal, resultant positive emotion may positively reinforce and promote additional weight-loss behaviors, making positive emotion take on a more erratic quality that depends on when the behaviors occur (Selby et al., 2014). On the other hand, on days where she fails to meet weight loss goals or regains weight, negative emotion may encourage weight-loss behaviors through negative reinforcement (Engel et al., 2013; Lavender et al., 2013). Thus, the synergistic effects between these two emotion processes likely contribute to the extreme weight loss seen in anorexia.

### What Drives Positive Emotional Instability in Anorexia Nervosa?

A growing body of literature has begun to link dysregulated positive emotion to symptoms of anorexia, but additional research is needed to elaborate the specific ways in which positive emotion regulation processes in anorexia contribute to positive emotional instability. Preliminary research has indicated that those with anorexia appear to exhibit deficits in positive emotion differentiation, indicating that many have difficulty distinguishing between positive emotions and tend to experience positive emotion in a global-diffuse manner (Selby et al., 2014). This experience of global-diffuse positive emotion may contribute to the reinforcement and motivation of weight-loss behaviors in anorexia, because the simple valence of various positive emotions (e.g., joy, happy) may promote positive reinforcement of anorexic behavior.

Additional factors may also contribute to positive emotional instability and dysregulation in anorexia. One potential cognitive factor may be positive rumination, which involves repetitively thinking about, reminiscing, or focusing on personal strengths or favorable life circumstances. Importantly, positive rumination is thought to be an active process that may lead to increases in positive emotion (Feldman et al., 2008). While positive rumination can have beneficial effects (Martin and Tesser, 1996; Larsen and Prizmic, 2004), in the case of anorexia, positive rumination about achieving weight-loss goals may facilitate motivation for weight-loss activities and striving toward weight-loss goals (Langens, 2002; Holmes et al., 2008). Imagining one’s “best possible self” (e.g., imagining attainment of the thin ideal) has also been found to predict immediate increases in positive emotion (Sheldon and Lyubomirsky, 2007). Planning weight-loss activities, calorie counting, and consumption of Pro-Anorexia quotes and images may also increase positive emotions, ultimately increasing engagement in weight-loss activities.

The role of positive emotions over the course of anorexia must be delineated as well. In the early stages of the disorder, it is possible that anorexia is precipitated by negative emotion, and weight-loss behaviors subsequently become coping mechanisms by providing positive emotion. On the other hand, negative emotion may become more prominent later, while the early stages of the disorder are characterized by more positive emotion arising from successful attainment of diet and exercise goals. For some, anorexia may even begin with the conception that improved body image or weight will lead to happiness. There may be important differences interactions between negative and positive emotion throughout the course of anorexia. Even after significant weight has been lost, for example, many with anorexia continue to lose weight. Recent research indicates that over-valuing happiness can be harmful, and may lead to feeling unhappy even when happiness seems just within reach (Mauss et al., 2011). Imagining future happiness associated with reaching a goal may eventually lead to disappointment when the goal is reached and associated happiness is less potent than originally imagined. Such may be the case with anorexia, where progress toward weight-loss goals may initially result in positive emotion, but upon reaching a larger goal may lead to continued disappointment (e.g., “I’m thinner, but still unhappy with my life”). This dissatisfaction may result in ever-increasing weight-loss goals in pursuit of an elusive happiness, where the pursuit of the goal results in more positive emotion than its attainment. In addition, later in the course of anorexia, weight-loss behaviors themselves may become inherently rewarding (Walsh, 2013; Selby et al., 2014). Alternatively, the later stages of the disorder may be primarily characterized by negative emotion and negative reinforcement, with positive emotion playing less of a role in maintaining the disorder. Clearly, more research is needed to clarify if, and how, positive and negative emotions dynamically interact with weight-loss behaviors over time.

The experience of positive emotion in anorexia must also be placed into broader cognitive and social contexts that go beyond the effects of weight-loss activities. Beliefs about happiness are shaped by the social environment, creating guidelines for how to achieve happiness and pursue positive feelings about the self. Current media often equates thinness with happiness by depicting thin people (particularly women) as flourishing in areas of their lives beyond ideal body appearance (e.g., career, wealth, romance, and family life). As a result, media sources may strengthen cognitive and implicit associations between thinness, happiness, and success in an unhealthy and overly broad way (Stice et al., 1994). Anorexic individuals may experience weight loss as success not only with a specific appearance goal, but in multiple other domains of life as well.

### Limitations

There were some limitations in this study. First, although we found evidence that the interaction between positive and negative emotional instability is related to increased weight-loss behaviors, causality cannot be established. Because emotional instability indices and weight-loss behaviors are calculated over the course of multiple days, the resultant picture is an intricate cycle where each may be partially responsible for the other. Previous research supports a bidirectional relationship, wherein increases in positive emotion both precede and follow weight-loss activities (Selby et al., 2014). Future studies should manipulate these factors experimentally to determine which predicts the other.

Second, the interaction between high levels of positive and negative emotional instability significantly predicted increased weight-loss behaviors, with the exception of self-induced vomiting. This finding, however, is not necessarily at odds with the rest, as self-induced vomiting is not only a weight-loss behavior, but also a very specific compensatory behavior that is often paired with binge eating episodes. Binge episodes have been strongly linked to the experience of negative emotional instability (Yu and Selby, 2013), potentially explaining this inconsistency. The combination of high positive and negative emotional instability did, however, predict increased self-induced vomiting compared to the combination of high positive and low negative emotional instability, partially supporting our hypothesis. Thus, while self-induced vomiting for the purpose of weight loss may be positively reinforced, negative emotion and negative reinforcement may primarily drive this behavior. Another alternative explanation for this finding may be that low positive emotion, or anhedonia, is more important for driving binge-purging behavior (Keating et al., 2012).

Third, use of aggregate indices for emotional instability also makes it impossible to determine the specific relationship between changes in experienced emotion and weight-loss behaviors. Future studies should examine specific emotional patterns in conjunction with discrete episodes of weight-loss behaviors, allowing for an in-depth examination of acute fluctuations in positive and negative emotion. Such studies could conduct an EMA monitoring period of a few days, and then use emotional instability indices generated from those data to predict weight loss activities reported with a subsequent EMA monitoring period or a longitudinal follow-up analysis in which weight loss behaviors are retrospectively reported. Such analyses would allow for establishing emotional instability as a risk predictor for weight loss activities.

Fourth, because all participants in this study were diagnosed with anorexia nervosa, we were unable to compare levels of emotional instability between those with anorexia to a non-eating disordered comparison group. Thus, it is unclear whether those with anorexia have higher levels of emotional instability than others with other mental health diagnoses or no mental health diagnosis. However, those with anorexia binge-purge subtype displayed higher levels of positive and negative emotional instability relative to those with anorexia restricting subtype, indicating that there are important differences in levels of emotional instability between those with anorexia, and the higher these levels are the more they are associated with weight loss activities. In addition, because this sample was primarily non-Hispanic Caucasian and did not include any men, these findings may not generalize to all individuals with anorexia. Finally, although the EMA methods used in the current study provide an incremental understanding of the positive and negative emotional experience in anorexia, and how those experiences may interface with weight loss behaviors, some important information about weight loss behaviors was not obtained. For example, future EMA studies assessing exercise sessions should have participants estimate the duration and intensity of the exercise session, as well as obtain a general estimate of how many calories were expended. Such information would provide a richer understanding of the type and nature of exercise sessions that women with anorexia engage in.

### Clinical Implications

Concomitant with front-line treatment of weight restoration, current treatments may be improved by better addressing the emotional experiences of those with anorexia (Wonderlich et al., 2014). In addition to reducing the intensity and instability of negative emotion in anorexia (Wilson et al., 2007), instability of positive emotion should be addressed in treatment, especially given this study’s finding that positive emotional instability was a strong predictor of weight-loss behaviors above and beyond mean-level negative emotional intensity. Helping patients understand the link between positive emotion and positive reinforcement of weight-loss activities using cognitive methods is one possible way to address this issue. Alternatively, it may be helpful to attempt to shift positive emotion behaviors from weight-loss activities to non-weight-related activities (Selby et al., 2014). Selecting alternative activities that provide a sense of reward as a function of effort may be particularly useful substitutes. Possible activities could include developing artistic or musical skills, or recreational activities such as equestrian activities, climbing, team sports, and/or balancing exercises (Duesund and Skårderud, 2003). Yoga may also be a viable alternative activity, with preliminary evidence already indicating that Yoga can reduce eating disorder symptoms beyond standard care (Carei et al., 2010). If physical activities are included in treatment, however, care should be taken to balance physical effort and exercise with appropriate nutritional intake (Zunker et al., 2011; Moola et al., 2013).

## Conclusion

Both negative and positive emotion, and negative and positive reinforcement, may be important factors contributing to the development and maintenance of anorexia. Neglecting the positive emotional experience of those with anorexia may constitute an incomplete approach to treatment. This study established the importance of positive emotion in anorexia, and highlighted the interaction between positive and negative emotional instability. Importantly, the interaction between positive and negative emotional instability predicted the highest level of weight-loss behaviors. Future research is needed to explicate the trajectory and intricacies of this relationship, and to examine further positive and negative reinforcement in anorexia.

### Conflict of Interest Statement

The authors declare that the research was conducted in the absence of any commercial or financial relationships that could be construed as a potential conflict of interest.

## Footnotes

1 The data for the present study were collection prior to the rise of smartphone apps utilized for EMA data collection, which are now being more commonly utilized.

2 An average report of approximately six exercise sessions over 2 weeks may at first seem to be a normal or healthy level of exercise, but it is important to consider that all participants in this study were diagnosed with anorexia nervosa, indicating severely reduced weight to an unhealthy level, and many participants were also in treatment—a major focus of which is weight restoration. Accordingly, six episodes of exercise has the potential to be quite unhealthy in this sample. For example, when many women with anorexia nervosa exercise, they will do so specifically with the goal of reducing calories consumed (Long and Smith, 1993). However, participants were not asked to estimate the intensity of their exercise or to estimate the amount of calories expended during exercise, limiting interpretations about the intensity or potential severity of exercise sessions in this sample.

3 Because these analyses utilized Poisson regression, interpretation of the B-weight value cannot be treated the same as in linear regression analyses, and magnitude of effects should instead be gaged by the RR statistic. Furthermore, although the value of this RR may seem small in comparison to the related Odds Ratio statistic, it is important to consider that the RR specifies an increase in the outcome variable for every one-unit increase in the independent variable, which makes interpretation of this statistic dependent on the scale of the independent variable. Because the positive and negative instability indices have large ranges of continuous scores, and the interaction is the multiplicative of these ranges, the number of units in the independent variable interaction term can range as high as 149,746.78. Thus, even a seemingly small 0.001 increase in relative risk represents a clinically significant outcome, and in the case of the present study a significant increase in reports of weight loss behaviors.
